# Supplementary material for: Serological Evidence of Influenza D Virus Circulation Among Cattle and Small Ruminants in France
Source: Viruses. 2019 Jun 5;11(6):516. doi: 10.3390/v11060516 (PMC6630579; doi:10.3390/v11060516)
Supplement: Supplementary file 1 [file viruses-11-00516-s001.pdf]

Table S1. Information available on sera tested, by region and by species

Region	Year of collecting	Bovine sera	Ovine sera	Caprine sera
Occitanie	2014-2018	1409 (31 herds)	960 (34 herds)	441 (10 herds)
Bretagne	2016	480 (27 herds)	164 (4 herds)	104 (2 herds)
Bourgogne-Franche-Comté	2017-2018	480 (20 herds)	-	-
Hauts-de-France	2014-2015	477 (6 herds)	306 (7 herds)	80 (1 herd)
Pays de la Loire	2015	480 (8 herds)	-	-

-: sera not available
